# Assessing Diabetic Polyneuropathy Prevalence in Hospitalized Patients: A Comparative Study Using the Michigan Self-Report Questionnaire and Physical Examination Methods

**DOI:** 10.1155/jdr/7027972

**Published:** 2025-06-08

**Authors:** Parastoo EsnaAshari, Elaheh Mianehsaz, Maryam Afarini, Mohammad Javad Azadchehr

**Affiliations:** ^1^Kashan University of Medical Sciences, Kashan, Iran; ^2^Department of Physical Medicine and Rehabilitation, Faculty of Medicine, Kashan University of Medical Sciences, Kashan, Iran; ^3^Department of Neurology, Faculty of Medicine, Kashan University of Medical Sciences, Kashan, Iran; ^4^Infectious Diseases Research Center, Kashan University of Medical Sciences, Kashan, Iran

**Keywords:** deep tendon reflex, diabetes mellitus, diabetic foot ulcer, diabetic neuropathies, physical examination, pressure, vibration

## Abstract

**Background:** Chronic complications of diabetes, such as diabetic polyneuropathy (DPN), are major contributors to disability and mortality among diabetic patients. Effective screening for DPN is crucial to prevent severe complications, including limb amputation. This study was aimed at assessing the prevalence of DPN among hospitalized diabetic patients using both self-report (Michigan questionnaire) and physical examination methods and at evaluating the level of agreement between these two screening approaches.

**Methods:** This cross-sectional study included 158 adult diabetic patients admitted to Shahid Beheshti Hospital in Kashan, Iran, between April and September 2023. Patients were screened for DPN using the Michigan self-report questionnaire and physical examination. Sociodemographic and clinical data were collected through patient interviews and medical records. Random sampling was used to ensure representativeness. Data analysis was conducted using SPSS Version 26, applying descriptive and inferential statistics. Cohen's kappa statistic was used to assess the level of agreement between the two screening methods. The validity and reliability of the assessment tools were confirmed through previous studies.

**Results:** The study found a moderate agreement between the physical examination and the Michigan self-report questionnaire in diagnosing DPN (*κ* = 0.486, *p* < 0.001). The overall prevalence of DPN, based on the agreement of both methods, was 64.6%. The prevalence was 75.9% based on physical examination and 72.8% based on the questionnaire.

**Conclusions:** This study found a high prevalence of DPN among hospitalized diabetic patients, highlighting the urgent need for effective screening methods. The moderate agreement between the Michigan questionnaire and physical examination suggests that the questionnaire could serve as a simple, cost-effective tool for early DPN detection. Further research and wider implementation of these screening tools may enhance early diagnosis and help reduce the risk of severe complications, such as limb amputation.

## 1. Introduction

According to the 2020 surveys by the International Diabetes Federation, 463 million people globally are affected by diabetes, including 55 million in the MENA (Middle East and North Africa) region, which encompasses Iran. By 2045, the number of people with diabetes is projected to increase to 108 million in the MENA region and 700 million globally.

The prevalence of diabetes in Iran almost doubled from 2007 to 2021 among adults 25 years old and above. Diabetes prevalence increased from 10.85% in 2016 to 14.15% in 2021. Prediabetes prevalence increased from 18.11% in 2016 to 24.81% in 2021 [1]. Diabetes prevalence in the MENA region, particularly Iran, is rising due to urbanization, dietary shifts, and sedentary lifestyles. Limited rural healthcare access, economic challenges, and an aging population exacerbate the issue, highlighting the need for prevention strategies and healthcare improvements [2]. Diabetic polyneuropathy (DPN) affects approximately 50% of long-term patients with Type 1 and Type 2 diabetes, with about 10% of patients already having neuropathy at the time of diabetes diagnosis [3, 4].

DPN primarily impacts sensory nerves and affects the hands and feet in a characteristic pattern named glove and sock distribution [5]. Loss of large nerve fibers results in impaired vibration perception, proprioceptive awareness (sense of body position), and diminished ankle reflexes, while loss of fine nerve fibers impairs pain perception, superficial touch sensation, and heat discrimination [6]. Symptoms of DPN include numbness, balance problems, pain, and tingling sensations. However, many patients remain asymptomatic despite significant sensory neuron damage [6, 7].

One of the most severe complications of DPN is the development of diabetic foot ulcers, which significantly impact patient outcomes. Up to 25% of diabetic patients will experience diabetic foot ulcers during their lifetime, a leading cause of hospitalization [8]. Patients with diabetic foot ulcers face increased risks of wound infection, amputation, and mortality [9]. The 5-year mortality rate for diabetic patients with foot ulcers is reported to be 40%, escalating to 63% for those undergoing amputation due to foot ulcers [10]. Inadequate screening and management of DPN can worsen chronic pain, negatively impacting quality of life, sleep patterns, and mental health [11, 12].

Early detection of DPN in hospitalized patients is often delayed due to a lack of standardized screening, the difficulty in recognizing subtle symptoms, and limited access to diagnostic tools. These challenges are further complicated by the high prevalence of comorbidities, especially among elderly patients [13]. A systematic review and meta-analysis encompassing 21 studies with 5540 diabetic patients in Iran found that the prevalence of DPN ranged from 16% to 87%, with an overall estimated prevalence of 53%. This indicates that more than half of diabetic patients in Iran are affected by DPN [14]. The combination of limited resources, staffing shortages, and infrastructural challenges in Iranian hospitals necessitates the use of straightforward and cost-effective screening tools for the early detection of DPN [15]. Early detection through accessible screening tests, alongside meticulous blood sugar control, patient education, and lifestyle modifications, is essential for preventing further complications [16]. By improving these practices, hospitals can significantly reduce the risks of advanced DPN, such as amputations, and enhance patient outcomes, ultimately lowering healthcare costs [17].

Although various diagnostic tools are available for screening DPN, gold-standard methods such as nerve conduction studies and intraepidermal nerve fiber density measurement (which assesses the number of nerve fibers in the outermost layer of the skin) are invasive, time-consuming, and typically restricted to research settings. Advanced techniques like SUDOSCAN, corneal confocal microscopy, and quantitative sensory testing also have limitations due to high costs, the need for specialized equipment, and trained personnel—making them less practical in resource-limited clinical environments [18–20]. In contrast, the Michigan Neuropathy Screening Instrument (MNSI), which includes both a questionnaire and physical examination, is low-cost, easy to administer, and suitable for routine clinical use in Iranian hospitals [21, 22]. The questionnaire allows for rapid symptom screening, while the physical exam offers more objective confirmation of neuropathic signs. Comparing these tools helps identify the most feasible and effective method for early detection of DPN, particularly in hospitalized settings with limited resources and trained staff.

The primary aim of this study is to investigate the prevalence of DPN among hospitalized diabetic patients. Additionally, this study will assess the effectiveness and level of agreement between two common screening tools—a self-administered questionnaire and physical examination—to determine their utility in clinical practice.

## 2. Materials and Methods

In this cross-sectional study (IR.KAUMS.MEDNT.REC.1400.042), diabetic patients over 18 years of age hospitalized in Shahid Beheshti Hospital in Kashan were examined with informed consent. Patients who met at least one of the following criteria were included in the study:

• a confirmed diagnosis of diabetes by a physician,
• current treatment with oral hypoglycemic agents or insulin as part of diabetes management, and
• fasting blood sugar (FBS) levels of 126 mg/dL or higher on at least two separate occasions, consistent with the diagnostic threshold for diabetes set by the American Diabetes Association guidelines [23].

Exclusion criteria: Patients were excluded if they had conditions that could confound the assessment of DPN, including

• Neurological diseases: hereditary or acquired neurological conditions, such as neuropathies due to chronic uremia or hypothyroidism, or a history of lumbar vertebrae or spinal cord damage.
• Autoimmune and vascular disorders: autoimmune diseases, collagen vascular diseases, and acute or chronic vasculitis.
• Other confounding conditions: cancer, varicose veins, and history of alcohol or neurotoxic drug use.

These exclusions were applied to minimize confounding factors that could independently influence neuropathy or mimic its symptoms, ensuring the reliability of the study findings on DPN [7].

### 2.1. Sample Size and Sampling Method

Using a 75.1% prevalence rate for DPN from the study by Janghorbani et al. [24] and considering a 95% confidence interval with an acceptable error of 7%, the required sample size was calculated to be 158 patients.

Patients were selected between April 2023 and September 2023 through twice-weekly visits to the hospital's inpatient wards. Based on the list of hospitalized diabetic patients in the relevant wards, a simple random sampling method was applied using computer-generated random numbers via SPSS (Version 26). If a selected patient did not meet the inclusion criteria, they were replaced with the next eligible patient in the randomized list.

### 2.2. Data Collection

Sociodemographic data, collected through face-to-face structured interviews, include age, gender, marital status, educational status, smoking history, history of diabetic ulcers, use of prescribed medical shoes or insoles, and physical activity level. Physical activity levels were categorized as regular (≥ 4 days/week), irregular (1–3 days/week), and inactive (< 1 day/week).

Clinical data were obtained from medical records and verified through patient interviews to ensure accuracy. Key variables, including the type and duration of diabetes, medication use, FBS levels, body mass index (BMI), and the presence of nephropathy, cardiovascular diseases, hypertension, and dyslipidemia, were meticulously reviewed. Additionally, pharmacy records and follow-up notes were cross-checked to validate essential information, such as diabetes duration and prescribed medications, ensuring the reliability of the data collected.

### 2.3. MNSI

The Michigan questionnaire comprised 15 questions assessing sensory symptoms such as numbness, burning pain, sensitivity to touch, tingling, itching, muscle weakness, cramps, water temperature detection, previous diagnosis of DPN, dry skin, history of diabetic ulcers, and history of amputation. The Neurology Department at Zahedan University of Medical Sciences has translated and validated the MNSI into Persian for use in Iran. The study confirmed that the Persian version is a reliable and accurate tool for diagnosing DPN in Iranian patients with Type 2 diabetes, ensuring its cultural and linguistic suitability for the target population [25].

The questionnaire was administered through interviewer-based assessments. Responses were scored based on the presence or absence of symptoms, with a score of four or more considered abnormal, indicating DPN. The scoring system for the MNSI questionnaire used a cutoff score of four or more, which was derived from prior validation studies. Specifically, Feldman et al. proposed this cutoff as part of a two-step quantitative assessment to diagnose and stage DPN effectively. Similarly, Khawaja et al. validated this threshold in a Jordanian cohort, further supporting its applicability across diverse populations with Type 2 diabetes [26, 27].

The MNSI physical examination involved the following steps:

• Visual inspection: Both feet were checked for deformities, dry skin, calluses, infections, fissures, and ulcers. Normal feet scored 0, while abnormalities scored 1. Ulcers were scored separately as 1.
• Achilles reflex test: Reflexes were tested using a reflex hammer. Reflexes were scored as 0 for normal, 0.5 for diminished or present only with the Jendrassik maneuver, and 1 for absent reflexes.
• Vibration perception: A 128-Hz tuning fork was used on the great toe. Scores were 0 if the patient sensed vibrations normally, 0.5 if the time to sense cessation exceeded 10 s, and 1 if no vibration was felt.
• Pressure sensation: A 10-g monofilament was applied at 10 points on each foot. Sensation at 8–10 points scored 0, 1–7 points scored 0.5, and no sensation scored 1.

The scores from both feet were combined, leading to a total score out of 10. A total score of > 2 was identified as the threshold for diagnosing DPN. This cutoff was validated using receiver operating characteristic (ROC) curve analysis, ensuring an optimal balance of sensitivity and specificity when compared to nerve conduction studies, including the study by Moghtaderi et al. [25, 26, 28].

In the study, the MNSI was administered with measures to reduce bias and inter-rater variability. One examiner conducted all questionnaire interviews, and another performed all physical exams. Both examiners completed a 1-week training course led by neurology and physical medicine professors. A pilot group of patients was examined under the supervision of assistant professors, and calibration sessions were held to ensure consistent scoring before the main study began.

### 2.4. Data Analysis

Data were analyzed using SPSS software Version 26. Descriptive statistics (frequency distribution and mean ± SD) and inferential statistics (Cohen's kappa statistic) were used to assess agreement between the self-report questionnaire and physical examination for the overall diagnosis of DPN.

## 3. Results

The study included 158 diabetic patients, with a higher proportion of male patients (52.5%). The mean age was 63.28 ± 14.29 years, and 66% were over 60 years old. Most patients (82.3%) had an education level of undergraduate or lower, and 94% had minimal physical activity (walking less than 1 day/week). Half of the patients were overweight.

Common comorbidities included hypertension (67.1%) and dyslipidemia (62%). The mean diabetes duration was 11.66 ± 8.31 years, with 66% having diabetes for 10 years or less. Type 2 diabetes was predominant (95.6%), and only 35.4% had controlled blood sugar levels. A detailed breakdown of the data is provided in Table 1.

### 3.1. Prevalence of DPN

According to Tables 2 and 3, the prevalence of DPN in patients studied through the Michigan self-report questionnaire and physical examination was 72.8% and 75.9%, respectively. The high prevalence of DPN in this hospitalized population highlights the necessity of regular screening in acute care settings. The slightly higher prevalence detected by physical examination (75.9%) compared to the self-report questionnaire (72.8%) suggests that physical examination may identify more cases, particularly in asymptomatic patients.

**Figure 1:**
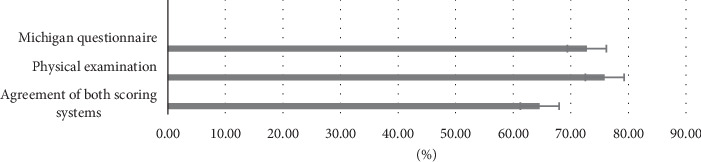
Prevalence of DPN across screening methods.

As illustrated in Figure 1, 64.6% of participants in this survey had DPN based on the agreement of both scoring systems.

### 3.2. Agreement Between Screening Methods for DPN Diagnosis

**Figure 2:**
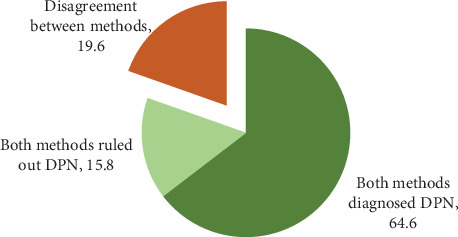
Agreement between screening methods for DPN diagnosis.

The results in Table 4 indicate that there is moderate agreement between the two physical examination scoring systems and the Michigan self-report questionnaire in the diagnosis of DPN (kappa = 0.486, *p* < 0.001). There was moderate agreement (95% CI: K = 0.41–0.60) between the two scoring systems in 80.4% (64.6 + 15.8) of cases showing the presence or absence of DPN (see Figure 2).

## 4. Discussion

The current study was aimed at determining the prevalence of DPN using two methods: the Michigan self-report questionnaire and a physical examination in hospitalized diabetic patients. Additionally, it aimed to assess the agreement between these two diagnostic tools.

This study is a cross-sectional study because it effectively assesses prevalence and compares screening tools. It allows for the simultaneous analysis of multiple variables, quickly identifies patterns, and is more cost-effective than cohort studies, which require extended follow-up.

### 4.1. Prevalence of DPN

In this survey, 64.6% of participants had DPN based on the agreement of both scoring systems. Previous studies in Iran reported varying DPN prevalence: For example, a study in Isfahan involving 810 diabetic patients reported a DPN prevalence of 75.1% [25]. Another study in Yazd, involving 2350 patients and using a combination of symptom examination and nerve conduction studies, found a prevalence rate of 51.7% [29]. Conversely, a study in Kashan using the monofilament test reported a DPN prevalence of 15.6% [30]. In neighboring countries, DPN rates were 39.5% in Jordan, 37.45% in Saudi Arabia, and 11.25% in Islamabad, all using the Michigan screening tool and ambulatory clinical screening in outpatient's population [27, 31, 32]. These disparities may result from differences in sampling methods, target populations, and screening techniques. Studies using multiple screening tools (e.g., MNSI, nerve conduction studies, and physical exams) reported higher prevalence rates, while outpatient studies relying on less sensitive tools (e.g., monofilament test alone) showed lower rates.

DPN presents with a range of symptoms, including positive (e.g., tingling and burning pain) and negative (e.g., numbness) sensory disturbances, which can lead to serious complications such as ulcers, infections, and amputations. In this study, the most frequently reported symptoms were sensitivity to touch (93%) and burning pain (83.5%), followed by prickling sensation (88.7%) and numbness (85.2%). Although DPN prevalence was lower in Jordan (39.5%, Khawaja et al.) and Islamabad (11.25%, Qureshi et al.) compared to this study, common symptoms across studies included burning pain, tingling, and numbness [27, 31]. These findings suggest that certain neuropathic symptoms are universally prevalent among diabetic patients, which are clinically significant as they indicate impaired protective sensation, increasing the risk of unnoticed injuries and foot ulceration. Emerging evidence emphasizes that painful neuropathic symptoms are predictive of diabetic foot complications, reinforcing the need for early detection and intervention to prevent severe outcomes [33].

The higher prevalence of DPN observed in this study may be attributed to its focus on hospitalized diabetic patients, who typically present with more advanced disease and comorbidities, as well as the use of both the MNSI questionnaire and physical examination. In contrast, outpatient studies often report lower prevalence rates, likely due to differences in sampling methods, patient populations, diabetes management, and the use of less sensitive screening tools such as the monofilament test alone. These disparities underscore the importance of standardized, comprehensive screening strategies and the need to consider regional and population-specific factors to ensure early and accurate detection of DPN.

In this study, hypertension (67.1%) and dyslipidemia (62%) were the most common comorbidities among participants. Studies have shown that hypertension and dyslipidemia worsen microvascular damage and oxidative stress, which are key mechanisms leading to the development and progression of DPN [7, 34]. Their high prevalence in the sample likely plays a significant role in the observed high DPN rates, emphasizing the importance of comprehensive cardiovascular risk management in diabetic patients.

The study found high rates of physical inactivity (93.7%) and overweight/obesity (72.8%) among participants—both recognized risk factors for DPN. These conditions contribute to poor glycemic control and systemic inflammation, accelerating nerve damage. Evidence shows that obesity and sedentary lifestyles increase DPN risk and severity, while lifestyle modifications like weight loss and increased activity can improve symptoms [34, 35]. Their high prevalence likely plays a key role in the elevated DPN rates observed in this study.

### 4.2. Diagnostic Tools for DPN

Nerve conduction studies and intraepidermal nerve fiber density measurement via skin punch biopsy are the gold standard for diagnosing DPN but are invasive, time-consuming, and require specialized equipment, limiting their routine clinical use [18–20]. Recent guidelines recommend simpler, noninvasive methods such as the 10 g monofilament test for protective sensation, vibration testing (tuning fork or biothesiometer) for large fiber function, foot inspections for structural or skin changes, and symptom assessments like the MNSI. These approaches are widely used due to their ease, cost-effectiveness, and reliability in detecting neuropathic changes [26].

In this study, the prevalence of DPN was assessed using two methods: physical examination and the Michigan self-report questionnaire. The MNSI was chosen due to its accessibility, cost-effectiveness, and ease of use in clinical practice. The MNSI, comprising a self-reported questionnaire and a physical examination, provides a comprehensive yet noninvasive assessment of neuropathic symptoms. Its reliability has been validated in various populations, including studies in Middle Eastern and Iranian cohorts, demonstrating strong sensitivity and specificity. Given its practicality, the MNSI is an effective screening tool for DPN, particularly in resource-limited settings [14, 26, 31]. The results showed a prevalence of 75.9% through physical examination and 72.8% using the Michigan questionnaire. Notably, there was a moderate agreement of 80.4% (95% CI: *K* = 0.41–0.60) between the two scoring systems, indicating a strong correlation between these diagnostic approaches.

In the Jimma study by Abdissa et al. [36], polyneuropathy prevalence among Type 2 diabetes patients was 53.6%. Using the Michigan self-report questionnaire alone, prevalence dropped to 18.9%, while the physical examination showed 51.1%. The Jimma study applied a stricter Michigan questionnaire cutoff (≥ 7) compared to Feldman et al.'s recommendation (≥ 4), likely reducing sensitivity and leading to a lower reported prevalence [27]. In contrast, this study found a higher prevalence: 75.9% via physical examination and 72.8% via the Michigan questionnaire. The use of the lower cutoff (≥ 4) aligns with previous research supporting better sensitivity for neuropathy detection. The higher prevalence in this study is likely due to a more sensitive diagnostic approach, differences in patient characteristics, clinical settings, and available resources. Additionally, the greater comorbidity burden among hospitalized patients may have contributed to the increased prevalence of DPN.

An ideal screening method for DPN should be easy to use, widely accessible, cost-effective, and provide acceptable accuracy. The Michigan questionnaire is particularly convenient in a hospital setting, as it allows for quick and simple screening. However, its reliance on patient self-reporting introduces limitations, such as subjectivity, recall bias, and the potential for overreporting or underreporting of symptoms. This issue is especially relevant for patients with cognitive impairments, low health literacy, or communication difficulties, who may struggle to accurately describe their symptoms. On the other hand, physical examination provides a more objective and comprehensive assessment, as it directly evaluates sensory function, reflexes, and neuropathic signs. However, it poses challenges in clinical practice, particularly in resource-limited settings, where access to monofilaments, reflex hammers, and tuning forks may be restricted. Additionally, performing an effective physical exam requires trained healthcare personnel, making it less feasible in overburdened healthcare systems or primary care settings where specialized expertise is lacking. Given the strong agreement between the Michigan questionnaire and physical examination, the Michigan questionnaire proves to be a practical screening tool for DPN in hospitalized patients. Its ease of use makes it particularly valuable in resource-limited or time-constrained settings.

This study provides a detailed analysis of DPN prevalence in hospitalized diabetic patients, a high-risk yet often underrepresented group. The findings emphasize the need for targeted screening in acute care settings to enable early detection and intervention, potentially preventing severe complications. Furthermore, this study is among the few to use both the Michigan questionnaire and physical examination for DPN diagnosis, providing a direct comparison of their agreement levels. This dual-method approach enhances diagnostic reliability and contributes to the discussion on effective screening tools for DPN.

### 4.3. Limitations

However, the study has limitations, including its single-center design, small sample size, and selection bias due to focusing solely on hospitalized diabetic patients. These factors may affect the generalizability of the findings in assessing the prevalence of DPN. For instance, the single-center design and small sample size limit the ability to generalize the results to a broader population, particularly to diabetic populations with different demographic or clinical characteristics. The reliance on self-report questionnaires, such as the MNSI, may also introduce response bias. Patients might underreport symptoms due to fear of additional treatments or overreport symptoms to receive more attention from healthcare providers. These biases could potentially affect the accuracy of the prevalence estimates.

Additionally, the cross-sectional design of the study limits the ability to assess changes over time and the progression of DPN. Future longitudinal and multicenter studies involving diverse diabetic populations and larger sample sizes are needed to validate and refine these screening methods. Such studies should also explore the impact of cultural and socioeconomic factors on DPN prevalence and screening accuracy.

## 5. Conclusion

The high prevalence of DPN among hospitalized diabetic patients underscores the urgent need for effective and accessible screening methods. The moderate concordance observed between the Michigan questionnaire and physical examination suggests that the questionnaire could serve as a cost-effective initial screening tool, particularly in resource-limited settings. Early detection and intervention are crucial to mitigate the risk of severe complications such as limb amputation.

Future research should focus on validating the Michigan questionnaire in diverse diabetic populations and exploring its integration into routine hospital protocols for early DPN detection. Longitudinal studies are also necessary to assess the progression of DPN and the long-term impact of early intervention strategies. Furthermore, future studies should include broader and more diverse samples to enhance the generalizability and applicability of the screening methods.

## Figures and Tables

**Table 1:** Sociodemographic, clinical, and laboratory data of the study participants.

**Variable**		**N** ** (%)**
Gender	Male	83 (52.5)
	Female	75 (47.5)

Age (year)	< 30	4 (2.5)
	30–40	7 (4.4)
	41–50	20 (12.7)
	51–60	23 (14.6)
	> 60	104 (65.8)

Working status	Employed	67 (42.4)
	Unemployed	75 (47.5)
	Retired	16 (10.1)

Education status	Illiterate	51 (32.3)
	High school	79 (50)
	Diploma	23 (14.6)
	Bachelor's degree	5 (3.1)

Physical activity	Inactive	148 (93.7)
	Irregular	7 (4.4)
	Regular	3 (1.9)

Body mass index (kg/m^2^)	Normal (18.5–24.9)	43 (27.2)
	Overweight (25–29.9)	79 (50)
	Obese (≥ 30)	36 (22.8)

Use of prescribed medical shoes		8 (5.1)

Smoking		19 (12)

Underlying disease	Hypertension	106 (67.1)
	Dyslipidemia	98 (62)
	Cardiovascular diseases	65 (41.1)

Type of diabetes	Type 1	7 (4.4)
	Type 2	151 (95.6)

Duration of diabetes (year)	< 5	22 (13.9)
	5–10	82 (51.9)
	11–15	17 (10.8)
	16–20	17 (10.8)
	> 20	20 (12.6)

Type of treatment	Oral hypoglycemic agents	83 (52.5)
	Insulin	74 (46.8)
	None	1 (0.6)

Controlled FBS (≤ 140)		56 (35.4)

**Table 2:** Frequency distribution of patients' responses to the Michigan self-report questionnaire.

**Symptom**	**N** ** (%)**
Are your legs and/or feet numb?	115 (72.8)
Do you ever have any burning pain in your legs and/or feet?	132 (83.5)
Are your feet too sensitive to touch?	150 (94.9)
Do you get muscle cramps in your legs and/or feet?	70 (44.3)
Do you ever have any prickling feelings in your legs and/or feet?	130 (82.3)
Does it hurt when the bed covers touch your skin?	16 (10.1)
Can you differentiate hot water from cold water in the tub/shower?	141 (89.2)
Have you ever had an open sore on your foot?	49 (31)
Has your doctor ever told you that you have neuropathy?	4 (2.5)
Do you feel weak all over most of the time?	114 (72.2)
Are your symptoms worse at night?	69 (43.7)
Do your legs/feet hurt when you walk?	89 (56.3)
Are you able to sense your feet when you walk?	147 (93)
Is the skin on your feet so dry that it cracks open?	81 (51.3)
Have you ever had an amputation?	5 (3.2)
Positive for DPN: 115 (72.8)	

**Table 3:** Findings of Michigan physical examination.

**Physical examination**	**N** ** (%)**
Appearance of feet	
Deformities	
Right	1 (0.6)
Left	0
Both	3 (1.9)
Dry skin/calluses	
Right	0
Left	0
Both	102 (64.6)
Infection	
Right	7 (4.4)
Left	7 (4.4)
Both	20 (12.7)
Fisher	
Right	0
Left	0
Both	38 (24.1)
Ulcer	
Right	15 (9.5)
Left	11 (7)
Both	13 (8.2)
Ankle reflexes	
Right	
Reduced	3 (1.9)
Lack	2 (1.3)
Left	
Reduced	12 (7.6)
Lack	2 (1.3)
Both	
Reduced	19 (12)
Lack	56 (35.4)
Reduced (right) and lack (left)	4 (2.5)
Reduced (left) and lack (right)	4 (2.5)
Vibration perception	
Right	
Reduced	3 (1.9)
Lack	2 (1.3)
Left	
Reduced	5 (3.2)
Lack	4 (2)
Both	
Reduced	17 (10.8)
Lack	89 (56.3)
Reduced (right) and lack (left)	2 (1.3)
Reduced (left) and lack (right)	0
Monofilament test	
Right	
Reduced	8 (5.1)
Lack	0
Left	
Reduced	4 (2.5)
Lack	1 (0.6)
Both	
Reduced	31 (19.6)
Lack	53 (33.5)
Reduced (right) and lack (left)	8 (5.1)
Reduced (left) and lack (right)	6 (3.8)
Positive for DPN: 120 (75.9)	

**Table 4:** Agreement between the physical examination and the Michigan self-report questionnaire in the diagnosis of diabetic neuropathy.

**Scoring system**		**Michigan self-report questionnaire**		**Kappa**	**p** ** value**
		**Positive**	**Negative**		
Michigan physical examination	Positive	102 (64.6)	18 (11.4)	0.486	< 0.001
	Negative	13 (8.2)	25 (15.8)		

## Data Availability

The datasets used and analyzed during the current study are available from the corresponding author upon reasonable request.
